# Leisure-time physical activity and its correlates in a multi-ethnic sample of adults over age 50 in Singapore

**DOI:** 10.1186/s12889-021-10431-6

**Published:** 2021-02-18

**Authors:** Joelle H. Fong

**Affiliations:** National University of Singapore, Postal: 469C Bukit Timah Road, Singapore, 259771 Singapore

**Keywords:** Physical activity, Exercise, Population aging, Retirement

## Abstract

**Background:**

To examine the prevalence and correlates of regular leisure-time physical activity (LTPA) among community-dwelling adults in Singapore.

**Methods:**

Data was sourced from the 2018–2019 Singapore Life Panel, which asked respondents about their current LTPA participation at various time-points over a seven-month period. The final sample comprised 7684 subjects over age 50. We applied logistic regression models in our analysis.

**Results:**

39.8% of the respondents engaged in regular LTPA, with significantly higher proportions of men than women doing so. Factors positively associated with regular LTPA in both genders were age, education, retired, income, and satisfaction with social life, whereas a negative association was found with self-rated health. The correlates of sustained LTPA participation were relatively consistent with factors predicting current participation.

**Conclusions:**

Regular participation in recreational physical activities is important to promoting health and well-being among middle-aged and older adults as populations age. Our findings indicated that positive perceptions of social relationships and being retired are important supporting factors. The urgent development of physical activity promotion strategies and interventions are required to foster greater overall LTPA participation.

## Background

Leisure-time physical activity (LTPA) has been defined as “physical activity performed during exercise, recreation or any time other than those associated with one’s regular occupation, housework, or transportation”, and is often distinguished from other domains of physical activity (e.g. occupational physical activity) in public health research [1]. Regular LTPA is defined as engaging in moderate or vigorous physical activity during leisure time for at least several times a week [2–4]. The surveillance of LTPA, including regular LTPA, among mid-age and older adults is particularly important since exercise can attenuate physiological declines associated with aging and disuse, and is associated with improved functional and mental health, lower risk of chronic diseases, subjective well-being, and better quality of life [5–11].

A substantial proportion of adults, however, do not participate in LTPA. This is especially so in some aging Asian societies [12–15]. An early study conducted in 1998 reports that 57.9% of persons aged 60+ in Singapore did not participate in regular LTPA [12]. More recent evidence indicates that, in around 2007–2008, the proportion of older adults reporting no LTPA/ exercise is 62.7% in Taiwan (PRC) and 67.7% in Shanghai, China [13, 14]. This contrasts with the lower levels of LTPA non-participation among mid-age and older persons that have been observed in the United States (20.8%), Switzerland (32.5%), Spain (30–45%), and England (46.5%) [16–19]. Policymakers and public health professionals in rapidly aging Asian societies have therefore focused intently on ways to promote active lifestyles and increase LTPA participation in this population segment.

Men tend to exhibit higher prevalence of LTPA participation compared to women [12, 15, 18]. Aside from gender, other correlates that have been found to be positively associated with LTPA include age, education, family encouragement, social support from friends, and awareness of the benefits of exercise [12, 13]. In addition, psychosocial indicators such as self-perceptions of positive social relationships, intrinsic motivation, and perceived social ties were correlated to higher LTPA involvement [20, 21]. Factors typically negatively associated with LTPA include number of chronic conditions, obesity, lack of time or company, having a job, worse self-rated health, and depression [12, 13, 18]. Fewer studies have examined correlates of LTPA participation in multi-ethnic populations. One exception is Tam et al. [22] which used a sample of 770 adults in Malaysia and found that physical activity levels were significantly related to ethnicity, gender, age, occupation, and educational level.

The current analysis explores the prevalence and correlates of regular LTPA participation in a large population-based sample of adults over age 50 in Singapore. Using 2018–2019 panel data, this present study seeks to (i) identify regular LTPA participation in January 2019, (ii) assess whether this health behavior was sustained over a period of time (specifically, 7 months), and (iii) examine the sociodemographic and health correlates of regular LTPA (measured both cross-sectionally and over 7 months). A city-state in Southeast Asia, Singapore will become a super-aged country by around 2024 where one in five persons will be age 65+ [23], and its residents are among the longest lived in the world. A Lancet study projects that average life expectancy at birth will increase from 83.3 years in 2016 to 85.4 years by 2040 in Singapore, placing it third highest out of the 195 countries evaluated [24]. Our study also fills an important gap in the literature by examining LTPA participation in a multi-ethnic population, and the panel design allows us to additionally explore whether factors predicting current participation are identical to those driving sustained participation.

## Methods

### Data

Data were from the Singapore Life Panel (SLP®), an ongoing high-frequency (monthly) internet survey administered for a representative cohort of Singaporean citizens and permanent residents aged between 50 and 75 when first recruited at baseline in July 2015. Since then, the SLP has followed more than 12,000 Singaporeans over 5 years, and monthly response rates have remained high at around 70% over time [25]. Comparisons between SLP and official statistics showed close matching on age, sex, marital status, ethnicity, education, labor force status, income and expenditure [25]. While the SLP is administered online for a majority of respondents, those who are unable to understand the survey questions or lacking internet access can answer the survey over the phone or at centres located conveniently around Singapore. Consequently, attrition rate is low. Ethical approval for the study was obtained by the Centre for Research on the Economics of Ageing in the Singapore Management University, and informed consent was obtained from each participant. A complete description of the SLP is given elsewhere [25].

To a large extent, the SLP was modeled after the American Life Panel and featured questions and survey techniques adopted from more established surveys like the US Health and Retirement Study [25]. The core SLP survey includes questions on spending, health, employment, socioeconomic status, retirement expectations, and social networks. Additional survey questions, including those on leisure-time and social activities, are fielded for a limited period in special modules. Specifically, we used data on leisure-time activities from the July 2018, October 2018, and January 2019 waves. Subjects with missing information on leisure-time activities were excluded. The final analytical sample used in this study comprised 7684 community-dwelling persons over age 50. It consisted of 47.3% males and 52.7% females. The average age among sampled respondents was 62.5 (SD = 6.0). Singapore is a multiracial and multicultural country, with ethnic Chinese making up the majority of the population. This was reflected in the sample: 87.2% were Chinese Singaporeans, 5.8% were Malays, 5.1% were Indians, and 1.8% were others (e.g. Eurasians). The survey questionnaire was available in all four official languages of Singapore — English, Mandarin, Malay and Tamil.

### Measurement of regular LTPA

Regular LTPA is defined as engaging in moderate and/or vigorous physical activity during leisure time for at least several times a week, which includes three or more times per week for vigorous activity or at least five times a week for light-moderate activities [2–4]. SLP respondents were asked to indicate their monthly frequency of participation in social/leisure physical activities such as exercises, swimming, and brisk walking. These are moderate- or vigorous-intensity activities that typically last at least 20 min each time. The survey did not feature questions about individual activity types. Response categories for overall LTPA engagement were “daily”, “several times per week”, “once a week”, “1-3 times a month”, or “less than once a month”. Using the January 2019 responses, we constructed a regular LTPA binary variable by combining the “daily” and “several times per week” responses into a “yes” category, and the other three responses into a “no” category. This was the main outcome variable of interest.

Data on LTPA were separately collated in July and October 2018 surveys from the same respondents. Thus in total, the sampled respondents’ LTPA participation was observed at three points in time over a seven-month period (July 2018, inclusive – January 2019). To explore whether factors predicting current participation are identical to those driving sustained participation in LTPA, we constructed an additional outcome variable (for sustained LTPA) coded 1 if the respondents reported regular LTPA participation at all three assessment points, and 0 otherwise.

### Measurement of covariates

Several correlates relating to LTPA were identified from previous studies [12–14, 18, 20, 21]. These were measured and defined as follows: demographic characteristics including age (50–54, 55–59, 60–64, 65–69, or ≥ 70), gender (male or female), ethnicity/race (Chinese, Malay, Indian or others), and current marital status (married or no); socioeconomic information such as schooling/highest educational qualification (less than secondary, secondary, and tertiary), employment (fully retired or no), living arrangement (live alone or with others), income (in logs), and number of children (units); as well as health and lifestyle factors such have activity of daily living (ADL) disability (yes or no), have chronic condition (yes or no), ever smoked (yes or no), worse self-rated health (yes or no), and whether satisfied with one’s social contacts and family life (yes or no).

The ADL disability measure referenced six items including difficulty dressing, walking across a room, bathing, eating, getting in or out of bed, and toileting (yes/no responses). The chronic condition measure was derived from the question: did a doctor tell you that you have any of the following conditions? (including hypertension, diabetes, cancer, heart problems, stroke, arthritis, and psychiatric problems; yes/no responses). An affirmative response to any one item was considered as having ADL disability and having chronic condition, respectively. Respondents reported their health status as excellent, very good, good, fair, and poor. The responses were dichotomized into fair/poor (worse self-rated health) or excellent/very good/good [18]. Survey participants were asked to indicate their current employment situation (working for pay, retired, self-employed, student, and others). These were non-mutually exclusive options. Those who stated retired and not working for pay or self-employed were considered fully retired.

Although less explored in previous studies, another potential confounder may be projected life expectancy. People who expect to live longer may be intrinsically more incentivized to participate in regular exercise. This factor is especially pertinent to Singapore’s context given the longevity of its residents as discussed earlier. Accordingly, we included a proxy measure (“expect to live past age 75”) was constructed from data on age and subjective life expectancy. For persons ≥ age 75, this indicator variable was set to 1 (< 1.5% of the sample). For persons < age 75, the indicator variable was coded 1 if the respondent stated 50% or more in reply to the question “What is the percent chance that you will live to be 75 or more?”, 0 otherwise.

### Statistical analyses

We analyzed men and women separately [12, 18]. Standard statistical *χ*^2^ tests (*α* = 0.05) for categorical data were conducted to determine if men and women significantly differ in their LTPA participation. Given a confirmatory result, we proceeded to analyze the prevalence and correlates of regular LTPA participation separately for men and women. First, we calculated descriptive measures for all variables of interest by gender. Second, we compared the reported prevalence for regular LTPA participation (January 2019 only) versus sustained participation over 7 months (July 2018 to January 2019). Third, we fitted logistic regression models by gender to assess factors independently associated for each dependent variable. Adjusted odds ratios (ORs) with their confidence intervals from multiple logistic regression models were reported. Statistical significance was established at *P* < .01 (two-tailed *P* values). All statistical analyses were carried out using STATA version 16.0 (StataCorp, College Station, TX, USA).

## Results

Table 1 shows the distribution of LTPA participation, sociodemographic characteristics, and health-related variables among our respondents. Of the 7684 respondents, 47.3% were men, 15.6% were aged 70 or older, 35.3% had tertiary education (more than 10 years of school), 79.2% were married, 21.7% were fully retired, and about a quarter (25.2%) lived alone. On average, annual income was $51,700 in Singapore dollars (approximately USD$36,000) and respondents had about two living children. Slightly more than a third (39.9%) of the sampled respondents rated their health as fair or poor, and 12.1% had ever smoked. Although 65.4% of the respondents reported having one or more chronic condition, only a small proportion (6.3%) had 1+ ADL limitation. More than half (58.3%) were currently satisfied with their social contacts and family life, and 22.4% expected to live/lived past age 75.

**Table 1:** Descriptive Statistics and Distribution of Leisure-Time Physical Activity: Singapore Life Panel, 2019

	Total, n (%)	Male, n (%)	Female, n (%)
	(***n*** = 7684)	(***n*** = 3632)	(***n*** = 4052)
Frequency of leisure-time physical activity/month			
Less than once	1595 (20.8)	671 (18.5)	924 (22.8)**
1–3 times	1463 (19.0)	669 (18.4)	794 (19.6)
Once a week	1571 (20.4)	745 (20.5)	826 (20.4)
Several times per week	1761 (22.9)	878 (24.2)	883 (21.8)*
Daily	1294 (16.8)	669 (18.4)	625 (15.4)**
Age group			
50–54	411 (5.3)	78 (2.1)	333 (8.2)
55–59	2379 (31.0)	1113 (30.6)	1266 (31.2)
60–64	2134 (27.8)	1030 (28.4)	1104 (27.2)
65–69	1560 (20.3)	783 (21.6)	777 (19.2)
≥ 70	1200 (15.6)	628 (17.3)	572 (14.1)
Race/ethnicity			
Chinese	6701 (87.2)	3167 (87.2)	3534 (87.2)
Malay	447 (5.8)	204 (5.6)	243 (6.0)
Indian	395 (5.1)	183 (5.0)	212 (5.2)
Others	141 (1.8)	78 (2.1)	63 (1.6)
Schooling			
Less than secondary	1781 (23.2)	678 (18.7)	1103 (27.2)
Secondary	3192 (41.5)	1390 (38.3)	1802 (44.5)
Tertiary	2711 (35.3)	1564 (43.1)	1147 (28.3)
Married	6084 (79.2)	3234 (89.0)	2850 (70.3)
Fully retired	1669 (21.7)	924 (25.4)	745 (18.4)
Lives alone	1935 (25.2)	863 (23.8)	1072 (26.5)
Income (in S$‘000) (SD)	51.7 (91.2)	58.5 (90.0)	45.6 (91.9)
Number of children (SD)	1.85 (0.99)	1.89 (0.96)	1.81 (1.02)
Worse self-rated health	3065 (39.9)	1443 (39.7)	1622 (40.0)
1+ ADL limitation	482 (6.3)	208 (5.7)	274 (6.8)
Have chronic condition(s)	5022 (65.4)	2464 (67.8)	2558 (63.1)
Ever smoke	928 (12.1)	798 (22.0)	130 (3.2)
Satisfied with social life	4477 (58.3)	2093 (57.6)	2384 (58.8)
Expect to live past age 75	1723 (22.4)	789 (21.7)	934 (23.1)

*P < .05; **P < .01 males vs. females. S$ refers to Singapore dollars (approximate 1S$ = USD$0.7)

About two-fifths (39.8%) of the respondents participated in regular LTPA, with significantly higher proportions of men than women doing so. Univariate analysis by individual response categories showed that significantly higher percentages of men than women exercised daily (*P* < .01) and several times a week (*P* < .05). Furthermore, on the flip side, significantly lower percentages of men than women exercised less than once a month (*P* < .05). Overall, this suggests that men and women in our sample differed in terms of their patterns of LTPA participation. Thus, the correlates of LTPA participation are analyzed separately for men and women. The distributions of sociodemographic and other covariates, except for smoking status, were generally quite similar among our male and female respondents.

Table 2 presents adjusted odds ratios for the likelihood of engaging in regular LTPA by gender. For men, variables significantly associated with LTPA participation were tertiary education, retired, income, satisfied with social life, worse self-rated health, age 70 and older, Malay, and 1+ ADL limitation. The magnitude of the estimated odds ratio for ‘fully retired’ was especially large as compared to the rest of the correlates, implying that transition into retirement is a key factor prompting LTPA participation among Singaporean men. Findings were similar for women, with the following exceptions: LTPA participation was also significantly associated with secondary education (*P* < .01), but it was not significantly associated with 1+ ADL limitation despite the estimated odds ratio being indicative of an inverse relationship with LTPA participation.

**Table 2:** Correlates of Regular  Leisure-Time Physical Activity

	Dependent Variable: LTPA in Jan 2019					
	Men			Women		
Independent Variable	OR		CI	OR		CI
Age group						
50–54 (ref)	1.00			1.00		
55–59	1.71	*	(1.03, 2.84)	1.08		(0.82, 1.42)
60–64	1.77	*	(1.07, 2.95)	1.28		(0.97, 1.70)
65–69	2.05	*	(1.19, 3.56)	1.40		(0.99, 1.97)
≥ 70	2.21	**	(1.24, 3.94)	2.20	***	(1.50, 3.23)
Race/ethnicity						
Chinese (ref)	1.00			1.00		
Malay	0.56	**	(0.40, 0.78)	0.52	***	(0.37, 0.72)
Indian	0.65	*	(0.46, 0.90)	0.76		(0.55, 1.03)
Others	1.29		(0.80, 2.08)	1.18		(0.70, 2.00)
Schooling						
Less than secondary (ref)	1.00			1.00		
Secondary	1.14		(0.93, 1.40)	1.29	**	(1.08, 1.53)
Tertiary	1.92	***	(1.56, 2.38)	1.85	***	(1.52, 2.26)
Married	0.94		(0.74, 1.21)	0.99		(0.84, 1.16)
Fully retired	2.26	***	(1.88, 2.72)	1.54	***	(1.28, 1.85)
Lives alone	0.99		(0.83, 1.18)	1.19	*	(1.01, 1.39)
Income	1.05	***	(1.03, 1.08)	1.04	***	(1.02, 1.06)
Number of children	0.95		(0.88, 1.04)	1.04		(0.97, 1.13)
Worse self-rated health	0.73	***	(0.62, 0.86)	0.68	***	(0.59, 0.8)
1+ ADL limitation	0.56	**	(0.40, 0.79)	0.93		(0.70, 1.22)
Have chronic condition(s)	0.90		(0.77, 1.05)	0.93		(0.80, 1.07)
Ever smoke	0.91		(0.76, 1.08)	1.87		(0.58, 1.27)
Satisfied with social life	1.44	***	(1.23, 1.69)	1.75	***	(1.51, 2.03)
Expect to live past age 75	1.10		(0.91, 1.33)	1.12		(0.94, 1.32)
*Model fit:*						
*Pseudo R2*	*8.0%*			*6.1%*		

Note: *OR* Odds ratio, *CI* Confidence interval

*P < .05; **P < .01; ***P < .001

Table 3 presents a comparative set of results from logistic regressions using LTPA participation sustained over a period of 7 months as the dependent variable (1769 out of 7684 respondents did so). It reveals that the correlates of sustained LTPA participation were relatively consistent with factors predicting current participation at a point in time. The pseudo R-squareds indicated an improved model fit over those obtained in Table 2 for both sexes. For men, however, a key difference was that 1+ ADL limitation was not significantly associated with LTPA sustained over time. Age was a strong and independent predictor of sustained regular exercise, controlling for other demographic and health factors. Women aged 65–69 and aged ≥70 were 2.03 and 3.51 times, respectively, as likely to adhere to regular LTPA as compared to their counterparts in the 50–54 age group.

**Table 3:** Correlates of Regular  Leisure-Time Physical Activity Sustained Over 7 Months

	Dependent Variable: LTPA (Jul 2018-Jan 2019)					
	Men			Women		
Independent Variable	OR		CI	OR		CI
Age group						
50–54 (ref)	1.00			1.00		
55–59	1.26		(0.69, 2.32)	1.04		(0.75, 1.46)
60–64	1.48		(0.81, 2.72)	1.26		(0.90, 1.77)
65–69	2.39	**	(1.26, 4.57)	2.03	**	(1.34, 3.05)
≥ 70	2.85	**	(1.45, 5.63)	3.51	***	(2.22, 5.55)
Race/ethnicity						
Chinese (ref)	1.00			1.00		
Malay	0.43	***	(0.27, 0.68)	0.44	***	(0.28, 0.70)
Indian	0.65	*	(0.44, 0.97)	0.66	*	(0.44, 0.98)
Others	1.59		(0.98, 2.57)	0.57		(0.29, 1.12)
Schooling						
Less than secondary (ref)	1.00			1.00		
Secondary	1.29		(0.99, 1.68)	1.55	***	(1.24, 1.93)
Tertiary	2.29	***	(1.76, 2.97)	2.18	***	(1.71, 2.79)
Married	0.84		(0.64, 1.11)	0.99		(0.82, 1.20)
Fully retired	2.41	***	(1.97, 2.95)	1.57	***	(1.27, 1.94)
Lives alone	0.93		(0.76, 1.14)	1.19		(0.99, 1.43)
Income	1.07	***	(1.04, 1.10)	1.04	**	(1.01, 1.07)
Number of children	0.92		(0.83, 1.01)	0.98		(0.90, 1.07)
Worse self-rated health	0.74	**	(0.61, 0.89)	0.66	***	(0.54, 0.79)
1+ ADL limitation	0.73		(0.48, 1.11)	1.01		(0.72, 1.43)
Have chronic condition(s)	1.04		(0.87, 1.24)	0.89		(0.75, 1.06)
Ever smoke	0.86		(0.70, 1.06)	0.82		(0.49, 1.38)
Satisfied with social life	1.72	***	(1.42, 2.07)	1.84	***	(1.53, 2.21)
Expect to live past age 75	1.18		(0.95, 1.47)	1.07		(0.87, 1.32)
*Model fit:*						
*Pseudo R2*	*10.5%*			*8.0%*		

Note: *OR* Odds ratio, *CI* Confidence interval

*P < .05; **P < .01; ***P < .001

## Discussion

Regular participation in recreational physical activities is important to promoting health and well-being among middle-aged and older adults as populations age. Yet, our study found that about three-fifths (60.2%) of Singaporeans over age 50 in a large community-based sample do not participate in regular LTPA. While direct cross-national comparisons cannot be undertaken given different study populations and methodology, this rate seems to be comparable to other aging Asian societies such as China, but higher compared to Western societies. Also worrisome is the fact that Singapore is transitioning quickly into a super-aged country where one in five of the residents will be aged 65 years and above. Although it was reported that 57.9% of elderly Singaporeans did not participate in regular LTPA in 1998 [8], the country was still relatively young then (share of population ≥ age 65 = 7.1%; average life expectancy at birth = 77.3 years). By contrast, the resident population is now demographically matured (in 2019: share of population ≥ age 65 = 16.0%; average life expectancy at birth = 83.6 years) [26].

Factors positively associated with LTPA participation in both genders were age, education, retired, income, and satisfaction with social life, whereas a negative association was found with self-rated health. These findings are generally consistent with those of previous research [9, 13], which showed higher age, higher education level, non-working, and better self-rated health, as important enabling factors. Our sensitivity analysis revealed that the correlates of sustained LTPA participation were relatively consistent with factors predicting current participation. While 1+ ADL limitation was predictive of a lower likelihood of engaging in exercise at a given time-point, it is not a determinant of longer-term adherence to regular LTPA.

The positive association between the likelihood of LTPA participation and being retired was salient in Singapore’s context because re-employment of older workers is encouraged up to age 67 (although statutory retirement age is 62). In 2019, the share of adults age 55–64 participating in the labor force is about 70%, with a higher labour force participation rate among men than women [27]. This rationalizes the comparatively large effect of ‘fully retired’ on LTPA participation in the regressions (particularly for men), as well as the positive association observed between LTPA participation and ≥ age 70. Thus, while aging societies seek to enhance the employability and retention of older workers in their (shrinking) workforce, the trade-off is that individuals who are still working are less likely to be physically active due to lack of time.

Our finding that satisfaction with social life was significantly associated with regular LTPA participation both cross-sectionally and over time (*P* < .001 in both cases) is consistent with emerging evidence that positive perceived social relationships and social ties are correlated to LTPA participation in the US and elsewhere [20, 21]. This has important implications in terms of public health interventions targeted at helping middle-aged and older adults develop positive attitudes on social interactions linked to their health behaviour.

Our results also indicated significant ethnic differences in regular LTPA participation, controlling for sociodemographic and health characteristics. Specifically, Singaporean Malays were less likely to engage in exercise than their Chinese peers (*P* < .001). This finding concurs with prior research which have investigated patterns of physical activity in multi-ethnic Asian urban populations [22, 28, 29]. One study, in particular, showed that elderly Chinese in multi-ethnic Malaysia were more physically active than their Indian and Malay counterparts [28]. Descriptive statistics from the Singapore Health 2012 study had also reported lower prevalence of regular LTPA (assessed using the Global Physical Activity Questionnaires) among Malay participants compared to other major ethnic groups [29]. Although our study did not look specifically at respondents’ reasons for not exercising, other studies have suggested that ethnic/ cultural norms related to physical activity (e.g., perceptions of whether it is a priority or desirable) may influence LTPA participation [22].

The strengths of this present study are its community-based, prospective design, and the sample is approximately three times larger than those employed in previous Singapore-based studies [12, 29]. We also adjusted for potential confounders such as satisfaction with social life, in addition to more conventional covariates. This study, however, has its limitations. First, the physical activity measure was self-reported and might be subject to recall bias or imprecise measurement. In our case, the fairly consistent LTPA responses elicited from each individual at different time-points provided some reassurance. Second, the data did not distinguish across different types of leisure physical activity nor include information about the intensity or duration of the activity. Finally, we examined sustained regular LTPA over a relatively short period of 7 months. Future research on LTPA covering longer follow-up periods and with richer measures on the different activity types and ethnic/ cultural norms will be required to investigate further the determinants of persistent LTPA participation.

## Conclusions

Lack of physical activity is a pressing public health issue worldwide, especially in rapidly aging Asian countries, where the lack of LTPA participation among middle-aged and older adults is most prevalent. Our study indicates that only about two-fifths of adults over age 50 in a large, multi-ethnic population-based sample in Singapore regularly engaged in LTPA in 2019. Factors associated with LTPA participation in both genders were age, ethnicity, education, fully retired, income, self-rated health, and satisfaction with social life. Physical activity promotion incorporating psychosocial intervention may help individuals develop positive attitudes on social interactions and social ties, which in turn, encourage LTPA participation. On cultural norms, more work needs to be done to understand potential differences in ethnic attitudes towards physical activity and to find ways of making LTPA more desirable for those who have the time and resources to participate. Policymakers need to evaluate the appropriate mix of strategies required to increase recreational physical activity levels in this demographic segment, and carefully weigh the health consequences of further increases in the statutory retirement age. Finally, efforts should be made to strengthen the monitoring of leisure activity engagement across various segments of adults, ranging from the middle-aged to the young-old to the oldest-old, so that effective lifestyle interventions can be more suitably developed and targeted at different population subgroups.

## Data Availability

The data that support the findings of this study are available from the Singapore Management University but restrictions apply to the availability of these data, which were used under license for the current study, and so are not publicly available. Data are however available from the authors upon reasonable request and with permission of the Singapore Management University. More information about the Singapore Life Panel can be found at (https://rosa.smu.edu.sg/singapore-monthly-panel).

## Abbreviations

ADL: Activities of daily living
LTPA: Leisure-time physical activity
SLP: Singapore Life Panel
